# Sustainability in Dutch and Flemish mental healthcare: A descriptive and comparative study

**DOI:** 10.1192/j.eurpsy.2023.1583

**Published:** 2023-07-19

**Authors:** K. Van Den Broeck, K. Catthoor, J. Luykx, M. De Hert, P. Niemegeers, H. Peeters, W. Krudop, J. Detraux

**Affiliations:** 1 FAMPOP / CAPRI, University of Antwerp, Antwerp, Belgium / Flemish Psychiatric Association, Wilrijk; 2 CAPRI, University of Antwerp / ZNA / Flemish Psychiatric Association, Antwerp, Belgium; 3 Dept. of Psychiatry & Neuropsychology, School for Mental Health and Neuroscience, Maastricht University Medical Center, Maastricht, Netherlands; 4 Dept. of Biomedical Sciences, Research Group Psychiatry, Center for Clinical Psychiatry / Antwerp Health Law and Ethics Chair, University Psychiatric Center KU Leuven / KU Leuven / AHLEC University of Antwerp, Leuven / Antwerp; 5 ZNA / Flemish Psychiatric Association, Antwerp; 6 University Hospitals Leuven, Leuven, Belgium; 7 St.-Antonius Hospital, Utrecht, Netherlands; 8 Dept. of Biomedical Sciences, Research Group Psychiatry, Public Health Psychiatry, KU Leuven, Leuven, Belgium

## Abstract

**Introduction:**

There is an urgent need for sustainable thinking and practices in healthcare systems to meet the challenge of climate change (Charlesworth & Jamieson, 2019; Corvalan et al., 2020; Luykx & Voetterl, 2022; Madden et al., 2020). This need is accelerated by the recent energy crisis. According to an international NGO policy paper (Karliner et al., 2019) healthcare institutions are large energy consumers and major emitters. The (mental) health sectors of the Netherlands and Flanders, the northern part of Belgium, also greatly contribute to the global climate crisis. Both regions have per capita emissions (between the 0.50t and 1t) that fall just below the world’s healthcare top emitters.

**Objectives:**

To evaluate the state of sustainability in Dutch and Flemish mental health institutions (including psychiatric hospitals, rehabilitation centers, and community mental health centers) and assess whether certain differences can be found in the climate policies of these institutions between both regions.

**Methods:**

Board members of mental health institutions were asked to complete a 20-item online survey in which concrete actions, objectives and ambitions in the field of sustainability were questioned. Frequencies and percentages were calculated for each question. For certain topics chi-squared tests were performed to test differences in sustainability issues addressed in the questionnaire between Dutch and Flemish mental healthcare institutions.

**Results:**

Survey response rates for Dutch and Flemish mental health institutions were 38% and 20%, respectively. Ninety-five percent and 38% of respectively the Dutch and Flemish institutions fully agreed that sustainability is a very important theme (χ2(1)=2,25, p=0,13). Key focus areas in both regions included sustainable energy transition (with half of the mental health institutions sourcing at least half of their energy via renewable energy resources and technologies) and recycling (almost 80% of the institutions). Statistically significant differences were found between both regions with regard to monitoring the environmental impact (Flanders 24% vs. The Netherlands 60%, χ2(1)=6,41, p=0,01) and fostering more sustainable commutes (Flanders 72% vs. The Netherlands 15%, χ2(1)=17,35, p<0,0001). The climate impact of medicines and food, as well as investments in sustainable projects, received little attention.

**Conclusions:**

Although a substantial part of Dutch and Flemish mental health institutions consider sustainability (very) important, a systemic ‘transformation’ will be necessary to make them climate neutral, as tenets of practicing mental healthcare sustainably include more than sustainable energy transition and recycling (Monsell et al., 2021). Moreover, a lack of sufficient investment opportunities, partly due to a lack of financial resources, seems to be the main barrier for many mental healthcare institutions for quickly reaching sustainability goals.

**Disclosure of Interest:**

None Declared

